# The Lack of Race and Ethnicity Data in Australia—A Threat to Achieving Health Equity

**DOI:** 10.3390/ijerph20085530

**Published:** 2023-04-17

**Authors:** Andre M. N. Renzaho

**Affiliations:** Translational Health Research Institute, School of Medicine, Campbelltown Campus, Western Sydney University, Locked Bag 1797, Penrith, NSW 2571, Australia; andre.renzaho@westernsydney.edu.au

**Keywords:** racial and ethnic disparities, health equity, invisible minorities, cultural groups

## Abstract

Collecting meaningful race and ethnicity data must be part of the national agenda and must be one of its primary objectives in order to achieve public good and support public interests. Yet, Australia does not collect data on race and ethnicity, and prefers the use of collective cultural groups, whose information is not consistently collected and reported at all levels of government and service delivery. This paper examines the current discrepancies in race and ethnicity data collection in Australia. The paper begins with examining the current practices related to collecting race and ethnicity data and then moves on to examine the various implications and public health significance of not collecting data on race and ethnicity in Australia. The evidence suggests that (1) race and ethnicity data matter, are imperative to ensuring proper advocacy and to reducing inequities in health and social determinant factors; (2) that White privilege is constructed as realized or unrealized personal and systemic racism; and (3) the use of non-committal collective terminologies makes visible minorities invisible, leads to the distorted allocation of governmental support, and legitimises and institutionalises racism and othering, hence perpetuating exclusion and the risk of victimisation. There is an urgent need for the collection of customized, culturally competent racial and ethnicity data that can be consistently integrated into all policy interventions, service delivery and research funding across all levels of governance in Australia. Reducing and eliminating racial and ethnic disparities is not only an ethical, social, and economic imperative, but must also be a critical item on the national agenda. Bridging the racial and ethnic disparities will require concerted whole-of-government efforts to collect consistent and reliable data that depict racial and ethnic characteristics beyond collective cultural groupings.

## 1. Introduction

Because of increased migration, most Organisation for Economic Co-operation and Development (OECD) countries have experienced increased cultural diversity since the 1970s, with 1 in 10 people in OECD countries being foreign born [1]. This is true in Australia, where data from the 2021 Australian census indicate that Australians have more than 300 different ancestral backgrounds, whilst half of Australians (48.2%) have a parent born overseas. More than a quarter (29.1%) are overseas-born migrants from over 190 different countries that speak over 400 languages at home [2]. This demographic profile ranks Australia sixth among OECD countries in terms of its intake of permanent migrants and second regarding the proportion of its population born overseas [1]. With increasing cultural pluralism in most OECD countries, reducing racial and ethnic disparities in terms of both health and social determinant factors is not only an ethical, social, and economic imperative, but it must also be a critical item on the national agenda [3]. Addressing racial and ethnic health disparities through systemic and institutional transformation cannot be achieved if adequate data on race and ethnicity are not collected. However, 20 out of 38 OECD countries, including France, Germany, Italy, Japan, and Denmark, do not collect racial or ethnic identity data [4]. 

The conflation, limitations, and confusion surrounding origins, ethnicity, race, nationality, and cultural attributes that have characterised census data in OECD countries, including Australia, over the last seven decades are well documented [5,6,7]. These studies have focused on comparative analyses of the changes over the last seven decades in the racial and ethnic classification schemes of censuses in Australia, Canada, and the United States, the historical analysis of the collection of taxonomic information related to diversity in the Commonwealth, and the appropriateness of the country of birth and the need to expand the ancestry variable as indicators of diversity [5,6,7]. None of these studies have examined the public health implications associated with the lack of an adequate collection of race and ethnicity data, which is the focus of the current paper. 

Nonetheless, race and ethnicity are two concepts that are closely related. Although controversial in terms of its operational definition and political correctness, race is rooted in the historical assumption that people can be divided into groups based on differences in their phenotypes and genetic makeup [8]. However, there is no evidence of a biological basis for identifying and classifying individuals into discrete racial groups [9]. Race is universally conceptualised as a social construct that permits the categorisation of people based on perceived physical differences [9]. Such physical traits may have social meaning ascribed to them and include facial features, complexion or skin colour, and hair texture. In contrast, ethnicity often refers to the grouping of people based on geography, group membership, and shared belief systems, such as religion, traditions and customs, language, heritage or ancestry [9]. Stevens and colleagues argue that racial and ethnic group membership that is assigned using rules of inheritance (physical traits), or through rules describing behaviours (e.g., spoken language), can be identified based on the choices and assignments made by individuals (self-identification) or can be shaped by the needs-based state or intuitional policies (e.g., routinely ascribed ethnic group information) that are geared towards reducing racial and ethnic disparities in health [5]. The methodological approaches used to collect race and ethnicity data are fundamental aspects of cultural competence, that is, improving the ability of service providers and service delivery systems to meet the needs of clients from diverse cultural, linguistic, and religious backgrounds; this also involves the ability of providers to improve clients’ access to services, their ability to competently navigate complex client–provider relationships and also their ability to navigate the larger and complex systems through which services are provided [10]. 

The opponents of measuring race and ethnicity contend that the categorisation of people based on differences in their biogenetically distinct traits leads to scientific or biological racism; this is where, historically, data from scientific research have been used to justify racism by highlighting the levels of racial inferiority or racial superiority in certain racial groups in health and social domains [8]. Hence, such views suggest that measuring race and ethnicity could be detrimental. Some scientists have refuted the biological explanation of racial disparities in health, arguing that race is a social construct and racial categorisations that emphasise genetic diversity in research are no longer considered scientific and should be phased out [8,11,12]. They argue this because of the available evidence that suggests that racial and ethnic disparities in disease burden disappear or diminish significantly once social, demographic, familial and economic characteristics, and the stage of diagnosis and type of therapy are controlled for in most studies [13,14,15,16,17,18]. Nonetheless, categorising race and ethnicity as social constructs could equally lead to cultural racism or differentialist racism [19]. This refers to shifting racist discourse from biological explanations to culturalist or nationalist classifications. In this sense, cultural racism encompasses culture-based prejudices and discrimination between ethnic or racial groups; this is where some cultures or ethnic groups could be portrayed as being superior (and thus also inferior) to others, or in other cases, they are seen as fundamentally incompatible and cannot co-exist in the same society [19].

While criticism of the assessment of race and ethnicity is beyond the scope of this paper, there is no doubt that collecting meaningful race and ethnicity data must be part of the national agenda and must be a primary objective in order to achieve public good and support public interests. Such objectives must be geared towards reducing racial and ethnic disparities in health, identifying and addressing new or evolving social determinants of health, countering racist practices or policies through the formulation of anti-racist strategies, enhancing inclusion and active civic participation, and informing effective resource allocation and interventions. For example, the coronavirus (COVID-19) pandemic highlighted the magnitude and impact of ethnic disparities in health at all levels of service delivery. Some countries, such as the United Kingdom (UK) [20,21] and the United States of America (USA) [22], implemented diverse analytical approaches during the COVID-19 pandemic to monitor and track changes regarding the impact of the disease; they reported race and ethnicity data in order to better understand the consistency in ethnicity and race data collection and coding across various administrative data sources. Such analyses shed light on how race and ethnicity affected COVID-19 outcomes. The studies noted a high level of agreement in the proportion of health records that stated patients’ ethnicity code before and during the COVID-19 pandemic across all administrative sources. The UK-based studies found that only 8.5% of inpatient records had a code of ‘not stated’ and that 8.8% had an ‘other ethnic group’ code, an increase from 6.1% and 7.2% since 2010, respectively [20,21]. In a USA-based study, the percentage of patients across all states with an “unknown” code for race and ethnicity at the beginning of the pandemic response averaged 29% and 39% for reported cases, and 15% and 29% for reported deaths, respectively. These figures decreased over time (i.e., demonstrating improvements in reporting race and ethnicity data) as the need to reduce racial and ethnic disparities evidenced in COVID-19 outcomes increased. However, despite the progress, there were significant problems related to the quality of and variations in the methods of data collection, which uses either routinely ascribed ethnic group information or Self-identification. Such variations have not been clear in reported race and ethnicity data, which makes it difficult to ascertain with certainty the risk of misclassifying race and ethnicity data. It is equally difficult to ascertain whether the missing data on race and ethnicity represent the individuals’ refusal to answer the question or can be accounted for by other factors (e.g., poor data processing and recording) [22]. Nonetheless, such complex levels of monitoring, tracking, evaluating the consistency of ethnicity and race data collection, and reporting specific race and ethnic disparities in health outcomes are not possible in Australia.

## 2. Levels of Data Collection: Censuses vs. Health Care System

The collection and reporting of data on race and ethnicity in censuses, as well as the guidelines that govern the operationalisation of race and ethnicity data, vary across countries (Table 1). In the USA, Canada, and the UK, the classification of race and ethnicity in censuses has focused on visible physical traits, especially skin colour, hair texture and facial features. The emphasis on skin colour is critical as it suggests that whiteness and privilege are interlinked. For the USA, the reported data tend to depict five categories for race (American Indian or Alaska Native, Asian, Black or African American, Native Hawaiian or Other Pacific Islander, White) and two categories for ethnicity (Hispanic or Latino and Not Hispanic or Latino) [23]. Such data can be analysed using single and distinct racial or ethnic groups, such as Black Hispanics, non-Hispanic Blacks, and non-Hispanic White, or broad racial or ethnic groupings, such as BIPOC (Black, Indigenous, People of Colour). Similarly, most health services in the USA collect race and ethnicity data. By 2008, more than 89% of hospitals and 87% of community health centres were collecting race and ethnicity data [24]. Recently, Polubriaginof and colleagues [25] examined the quality of race and ethnicity data using data from the Healthcare Cost and Utilization Project and the Optum Labs Data Warehouse (n = 165,975,722), and found that 25.3% and 26.0%, respectively, did not have informative data on race and ethnicity. When the authors extended their analyses to a single institution, the race and ethnicity data quality did not improve over time, with the loss of information occurring in 35.8% of updates. The reason for this was that race and ethnicity data are collected haphazardly. 

In Canada, by using mark-in circles and write-in spaces, there are 13 derived racial and ethnic groups, as well as the Indigenous status. The emphasis is on depicting ‘visible minority’ and population groups, including the following: (1) South Asian (e.g., Bangladeshi, Indian, Pakistani, Punjabi or Sri Lankan), (2) Chinese, (3) Black (e.g., African American, Congolese, Haitian or Nigerian), (4) Filipino, (5) Arab, (6) Latin American, (7) Southeast Asian (e.g., Vietnamese, Cambodian, Laotian, or Thai), (8) West Asian (e.g., Iranian or Afghan), (9) Korean, (10) Japanese, (11) other population groups that cannot be included elsewhere (e.g., Guyanese, Pacific Islanders, Polynesian, Tibetan, or West Indian), (12) multiple visible minorities (two or more mark-in responses associated with groups designated as visible minorities), (13) not a visible minority (i.e., White), and Indigenous status (First Nations, Métis or Inuk/Inuit) [27]. However, whilst ethnicity and race data are collected at the census level, they are seldom collected in hospitals and outpatient health services, making it hard to find complete administrative on ethnicity and race. Consequently, the Canadian Institute for Health Information has developed race-based and Indigenous identity standards, and has formulated associated guidance on the use of the standards for race-based and Indigenous identity data collection [9].

In the United Kingdom [28], the national census (England and Wales, Northern Ireland, and Scotland) and identity codes (codes used by the British police) report race and ethnicity data. Such data can be analysed using single and distinct racial groups, such as Asian or Asian British (i.e., Indian, Pakistani, Bangladeshi, Chinese, and any other Asian background), Black, Black British, Caribbean or African, mixed or multiple ethnic groups (i.e., White and Black Caribbean, White and Black African, or White), and White people, or by using broad racial and ethnic groupings, such as BAME (Black, Asian and Minority Ethnic). However, across all three administrative data sources (General Practice Extraction Service Data for Pandemic Planning and Research, Hospital Episode Statistics, and Improving Access to Psychological Therapies), the quality and completeness of ethnicity data vary; and data users do not always have access to the same data sources, hence increasing the risk of biased estimates [20,21]. Nonetheless, the proportion of health records that state the patient’s ethnicity code is very high (83.0–87%), and across all sources, the overall agreement rates are high and similar (89.0–93.0%) [20,21].

In Australia, the government is reluctant to report race and ethnicity data, and the emphasis is on variables that broadly measure cultural and language diversity (collective cultural groupings) and that are based on an individual’s country of birth and language spoken at home. These variables do not necessarily measure race and ethnicity, and their groupings depict an individual’s country or region of birth (e.g., Australia vs. other countries or Australian born vs. overseas born) or linguistic differentiation (e.g., English vs. other languages or English speaking vs. non-English speaking), often all packaged under the “Culturally and Linguistically Diverse (CALD)” terminology. In addition, the collection and reporting of data that inform the collective cultural groupings remain inconsistent at all levels of governance (e.g., national censuses vs. state-level surveys vs. service delivery), making it difficult to compare the effectiveness of programs in reducing racial and ethnic disparities in terms of health and social determinant factors.

## 3. Race and Ethnicity Data Matter and Are Imperative to Ensuring Proper Advocacy and Reducing Inequities in Health

Racial and ethnic disparities that are related to individuals’ disease burden (morbidity, mortality, financial cost, and disability-adjusted life years), response to treatment, and the quality of and access to care are well documented [29,30], with some racial and ethnic groups more affected [31,32,33]. For example, the call for closing the Indigenous health gap in North America, Canada, and Australia is premised upon the evidence that racial and ethnic health inequities seem to be rooted in socio-economic disadvantages that are linked with race and ethnic identity, and are associated with racism and stereotyping. 

### 3.1. The White Privilege as a Barrier and the Main Issue

From a social construction point of view, the biological explanation (racial superiority) for White privilege is weak, because race does not reflect a biological or genetic reality. It is instead a constructed reality of social hierarchy [34]. White people, who have propounded the group’s supremacy and have constructed racism as an abstract concept, have historically considered racism to be the consequence of the social construction of race, which, in turn, has subsequently degraded racial and ethnic minorities [34]. Such views discount the societal, systemic, institutional, and political structures that both overtly and inherently subjugate racial and ethnic minorities in order to protect White privilege [34]. Hence, White privilege has more to do with the advantages that White people have, such as economic, political, and educational privileges, that are unavailable to others [35]. There is no doubt that power and its associations with enhanced political influence and institutional policies, better education, and higher income facilitates access to good health, but access to such systems and institutional structures is restricted for racial and ethnic minorities.

That is why White privilege is considered as realized or unrealized personal and systemic racism [35]. In calling for action to address White privilege, Hobbs noted that White people’s power in terms of health care, and its association with personal and institutional privileges and opportunities, allows them to occupy privileged positions that perpetuate values and maintain services that are geared towards meeting the needs of the majority at the expense of racial and ethnic minorities. Such power and institutional privileges are critical in either sustaining the privilege gaps regarding race that exist in health care or to transforming health care systems in order to effectively reduce racial health gaps [36]. Where there is a will, achieving the latter is possible, given that the evidence points to racial health gaps not being accounted for in terms of the appropriateness of clinical interventions, needs, and patient preferences. Instead these discrepancies are explained by the racial privilege gap, and thus by ingrained discrimination at the personal, institutional, and societal levels [37]. In order to reduce the racial and ethnic disparities that exist in health, the social and economic problems (e.g., unequal distribution of power, money, and resources) that fuel inequalities and that are unfair and avoidable must first be tackled.

### 3.2. The Use of Non-Committal Collective Terminologies Makes Visible Minorities Invisible

The Indigenous health gap and its underlying causes are well documented in Australia, as the government collects data on Indigenous status (an approximately accurate measure of race and ethnicity) [29]. Such evidence illustrates what health and social interventions are needed for Indigenous people, and the potential for very large health and societal gains. However, apart from Indigenous status, Australia reports data on cultural diversity based on individuals’ country of birth and language spoken at home rather than explicit data on race and ethnicity. The use of the CALD terminology as a proxy measure of race and ethnicity is problematic and has some serious implications. For example, data on COVID-19 produced by the Australian Bureau of Statistics indicate that the COVID-19 burden, testing rates, and vaccination uptake disproportionally affected CALD communities. The age-standardised COVID-19 death rate among overseas-born Australians was three times that of Australian-born people (6.8 deaths per 100,000 people versus 2.3 deaths), with the highest age-standardised death rate recorded for migrants from the Middle East (29.3 deaths per 100,000 people). In contrast, the age-standardised death rate recorded for Sub-Saharan African-born Australians was among the lowest (3.4 deaths per 100,000 people), along with North-East Asian-born Australians (2.9 deaths per 100,000 people), United Kingdom and Ireland-born Australians (2.1 deaths per 100,000 people), and other North-West European-born Australians (3.1 deaths per 100,000 people). 

Broad aggregations of cultural groups can lead to false assumptions and the inadequate allocation of resources. For example, what percentage of the findings on Sub-Saharan African-born Austrialians in the COVID-19 death data above could be explained by White privilege? The 2021 census enumerated a total of 372,151 Australian residents who were born in Sub-Saharan Africa [38]. Of these, 189,207 (50.84%) were migrants from South Africa and 6847 (1.84%) were migrants from Zimbabwe. South African and Zimbabwe-born migrants who have moved to Australia are predominantly White South Africans (Afrikaner and British descent) and White Zimbabweans (English and Scottish), who are socio-demographically, economically, and ethnically different from their black African Australian counterparts. Broad aggregations of cultural groups also occur when discussing the Middle Eastern region, which is made of countries with people of different racial and ethnic backgrounds. For example, the Australian Government’s Department of Home Affairs’ data show huge racial and ethnic diversity among Lebanese (e.g., Maronite, Melkite, Orthodox Christians, Arabs, and Australians), Iraqi (e.g., Kurds, Assyrians, Chaldeans, Mandaeans and Armenians), Iranian (Iranians, Afghans, and Kurdish), Syrian (e.g., Syrians, Assyrians, and Arabs), Saudi Arabian (e.g., Arabs and Indians) or Omani (Arabs, Baluchi, and South Asian and African ethnic groups) migrants. Packaging these racially and ethnically different groups into one broad collective cultural group runs the risk of widening, rather than reducing, inequities.

### 3.3. The Use of Non-Committal Collective Terminologies Leads to Distorted Allocation of Funds

Relying on aggregated cultural diversity data rather than explicit data on race and ethnicity can lead to false assumptions, which is associated with a distorted allocation of funds. The inconsistent collection of data at all levels of governance, as occurs in the case of Australia, leads to poor racial and ethnic data mapping and the poor integration and co-ordination of efforts. It represents a missed opportunity to leverage information across all levels of government, which is caused by inconsistent measures that preclude a holistic view of racial and ethnic disparities and the effective equity-informed allocation of scarce resources. It fuels the under-estimation of health issues among racial and ethnic groups based on false assumptions, which can widen health inequities.

As an illustration of the danger associated with using non-committal cultural groupings, when reporting on the health of migrants, even the most trusted government departments [39] and organisations, such as the Australian Institute of Health and Welfare [40] and the Australian Bureau of Statistics [41], continue to highlight the ‘healthy migrant effect’. The main argument is that the pre-departure exclusion of unhealthy migrants due to the rigorous health screening that prospective skilled migrants are subjected to before entry reduces the financial burden on the Australian healthcare system (i.e., these migrants will use less healthcare than Australians on average). Hence, only those who are healthy are granted entry to Australia. In addition, skilled migration favours those who are resilient, meaning that ambitious and privileged healthy migrants who can financially and psychologically afford to relocate to another new country are more likely to apply for migration. These migrants tend to be highly skilled, educated and younger, all of which are social determinants of health. The blanket belief in the notion of the ‘healthy migrant effect’ fails to consider cultural factors and the health of forced migrants, such as refugees and asylum seekers, who remain invisible minorities. The findings that suggest migrants from the Family and Skill streams use less healthcare than Australians, as an illustration of the healthy migrant effect [39], may be misleading for many reasons. Access to and the utilisation of health services among these migrants are inextricably linked to race and ethnicity, and could be influenced by cultural factors that are often not accounted for in analyses (e.g., the underutilisation of health services due to cultural beliefs can be related to disease and health-seeking behaviours, unfamiliarity with the new health system, racism and the lack of cultural competence among health providers, past personal experiences with and an associated distrust of the health system, religious factors, the fear of the unknown, and so forth). In addition, refugees tend to come from specific countries torn by wars and armed conflicts. Yet, securing refugee status is also inextricably linked to race and ethnicity at every stage of the displacement cycle, by virtue of the cause of displacement (e.g., racial and ethnic minorities targeted during atrocities), the racial discrimination that accompanies their processing in transit countries, and the geopolitical interests of resettlement countries. Capturing refugees from war-torn countries under the broad and culturally collective “CALD” umbrella makes these visible minorities invisible. Such collective labelling does not depict race and ethnic characteristics, which can mask or even widen disparities. 

Additionally, there is the issue of ‘the salmon bias hypothesis’, where critically ill migrants choose to remigrate to their countries of birth to either convalesce and/or possibly die and be buried in their ancestral lands [42]. Consequently, the return of critically ill migrants to their countries of birth distorts the numerator (a numerator–denominator mismatch), and hence underestimates the health outcomes among certain migrant groups. This bias can only be properly captured if data on race and ethnicity are captured because remigration is closely linked to race and ethnicity, with certain racial and ethnic groups, such as Mexican and Hispanic people, being more likely to remigrate than others [43]. In England and Wales, among migrants from India, Pakistan, and Bangladesh, the likelihood of individuals remigrating to the countries of their birth increases after 55 years for males, but increases steadily over time for females. In contrast, remigration is highest among young adults for both males and females, but decreases steeply over time for migrants from the United States, Australia, Canada, New Zealand, and non-European Union countries; meanwhile, it decreases gradually for migrants from the European Union and Sub-Saharan Africa. However, results across studies have been inconsistent, with some studies reporting varying levels of remigration biases; this alone can either contribute but not sufficiently explain the advantages of migrants in terms of mortality and morbidity in some migrant groups, or explain the migrant mortality and morbidity advantage [44]. These inconsistencies are a result of differing study designs and the varying quality of data on remigration, as well as varied operational definitions of ethnicity. 

### 3.4. The Use of Non-Committal Collective Terminologies Legitimises and Institutionalises Racism 

The COVID-19 police crackdown in Sydney in 2021 created the ‘east–west double standard’. In the local government areas of South-West Sydney (Fairfield, Canterbury-Bankstown, and Liverpool), a region known to be multicultural, there was a COVID-19 police operation that was widely criticised as being heavy-handed and as displaying the over-policing of multicultural communities, which was interpreted as thinly veiled racism. In contrast, in the eastern suburbs, the COVID-19 police crackdown was accommodating, and beachgoers flocked to the beaches of the eastern suburbs without consequences, even though these local government areas were the origin of the outbreaks that spilled over to the South-West. It is interesting that the police operations were labelled as a ‘race’ issue without commensurate data on race and ethnicity in order to identify and protect the most vulnerable and invisible minorities, or monitor and address police racial profiling. Identical issues were evident in the low levels of COVID-19 vaccination uptake, low COVID-19 testing rates, and low levels of compliance with lockdown restrictions among multicultural communities. The lack of race and ethnicity data led to a blame game, with the invisible ethnic minorities being victimised rather than protected. In other instances, it has not been uncommon for crimes committed by some South Sudanese Australians to be generalised to all African communities under the umbrella of ‘African gangs’ [45]. Such a misplaced racist generalisation is legitimised and institutionalised in the absence of data on race and ethnicity.

### 3.5. Othering Perpetuate Exclusion and Risk of Victimisation

To prevent the risk of “othering” some advocates might argue that using collective groupings such as “CALD” terminologies increases the cell size and statistical power of this group. However, it masks invisible minorities, because most of these groupings include visible minorities who would not otherwise be identified by such approaches. As stated earlier, the “sub-Saharan African” category in most Australian statistics is predominantly made of White South Africans and Zimbabweans, and does not reflect the needs of the visible black African Australian minorities. Whilst the process of hegemonically ascribing otherness explicitly (due to small size) or implicitly (making visible minorities invisible in collective cultural groupings) to social groups may make them essentially different, invisible, otherwise inferior or not worth reporting in the context of research and policy formulation, it can also perpetuate prejudice and discrimination [46]. A typical example of this in Australia are initiatives and policies supposed to reduce gender inequalities, which do not take into account intersectionality and do not report data that depict race and ethnicity-related gender inequalities [47]. The lack of intersectionality in the gender equality debate and in racial and ethnicity data makes the current approach to reducing gender inequalities a White woman vs. a White man issue, even though the gender gap is increasingly a racial problem. This is an example of exclusionary othering, that is, the activation of power within relationships or decision-making that explicitly or implicitly emphasises domination and subordination, the consequence of which may include exclusion and marginalisation, alienation, internalized oppression, and decreased opportunities [48]. To address racial and ethnic disparities, inclusionary othering, that is, the activation of power within relationships or decision-making processes used to transform communities and build coalitions [48], must be a priority. The emphasis must be on the raising of coconsciousness in order to create a sense of community, inclusion and belonging; this is achieved by activating power within relationships and various systems in order to identify visible minorities using more inclusive and helpful categories based on race and ethnicity. Similarly, data from the COVID-19 pandemic in Australia and other industrialised nations point to ethnic minorities being disproportionately affected by the pandemic (higher risk of COVID-19 infections, more serious illnesses, more intensive care admissions, and higher mortality than the host population), yet they were excluded or underrepresented in major COVID-19 interventions, policies, and service delivery [49]. This under-representation or invisibility has been associated with the unequal distribution of resources and intervention rollout strategies [50]. Further complicating this, in the case of Australia, racial and ethnic minorities are heterogeneous groups, and their health risks, burdens, and outcomes differ across and within cultural groups and by country of birth, hence necessitating clear data on race and ethnicity. Whilst the COVID-19 pandemic highlighted the ethnic minority-dependent nature of the national economy, programs for racial and ethnic minorities are unfortunately virtually invisible in Australia, with research into racial and ethnic minorities’ health being grossly underfunded and receiving only 5% of all people-focused health research funding [51]. 

## 4. Conclusions

The available evidence calls for the collection of customized, culturally competent racial and ethnicity data in Australia that can be consistently integrated into interventions, service delivery, and research funding at all levels of governance. Currently, Australia is falling behind other OECD countries in tracking and addressing racial and ethnic inequities in health and social determinant factors. The UK and USA have robust systems for capturing race and ethnicity data at various levels of service delivery in order to detect and address inequities. For example, the UK Research and Innovation agency and the National Institute for Health and Care Research have developed policies to address under-representation and promote active participation at all levels of research funding scheme and academic rank, which they update regularly. Using harmonised race and ethnicity data, these institutions track inequities in research funding for both applicants and awardees, covering grants, fellowships, and PhD scholarships. Recently, the UK Office for National Statistics began reporting on the race and ethnicity pay gap each year and recommended mandatory ethnic pay gap reporting. The USA (US Department of Health and Human Services and the Office of Management and Budget) and Canada (Employment Equity Act) have clearly developed policy statements and protocols related to the inclusion of race and ethnicity in all data collection and reporting at all levels of government and service delivery. Such data are used to monitor inequities in research funding, to improve services by identifying and serving visible minority groups, to maintain quality improvement efforts, and to monitor progress towards reducing racial and ethnic disparities in health and social services. In these countries, government research funding institutions mandate the collection and reporting of individual-level data on race and ethnicity in order to monitor coverage, reach, and inclusion. In order to reduce racial and ethnic disparities in Australia, collecting data on race and ethnicity beyond collective cultural groupings is imperative. Bridging the racial and ethnic disparities will require concerted whole-of-government efforts in order to collect consistent and reliable data that depict racial and ethnic characteristics. Further studies are required to examine the best ways in which to operationalize race and ethnicity data in the Australian context in a meaningful way, and to examine the extent to which such data can be harmoniously and accurately recorded in different administrative databases at all levels of service delivery.

## Figures and Tables

**Table 1:** Operationalisation of race and ethnicity data in censuses in Australia, Canada, the United Kingdom, and the United States of America.

Variable	Modules	Output Categories or Grouping
Australia [26]		
Country of Birth Standard	Country of Birth of Person Country of Birth of Father, and Country of Birth of Mother	Australia vs. Others, recoded as born in Australia vs. Born overseas.
Language Standards	First Language Spoken Languages Spoken at Home Main Language Other Than English Spoken at Home Main Language Spoken at Home, and Proficiency in Spoken English.	English vs. Other language English Proficiency (Well or Very well, Not well or Not at all, Not stated/Inadequately described, and Not applicable)
Indigenous Status	Self-identification of Indigenous status	Neither Aboriginal nor Torres Strait Islander Origin Aboriginal but not Torres Strait Islander Origin Torres Strait Islander but not Aboriginal Origin Both Aboriginal and Torres Strait Islander Origin
Ancestry Standard	Cultural and ethnic origin groups broadly based on country of origin	Broad regional grouping e.g., Oceanian vs. North-West European vs. Southern and Eastern European vs. Sub-Saharan African
Canada [27]		
Self-identified race and ethnicity	Visible minority variable * and the population group variable based on mark-in circles and write-in spaces Thirteen derived groups and Indigenous status	South Asian (e.g., Bangladeshi, Indian, Pakistani, Punjabi or Sri Lankan) Chinese Black (e.g., write-in responses such as African American, Congolese, Haitian or Nigerian). Filipino Arab Latin American Southeast Asian West Asian Korean Japanese Other population groups not included elsewhere (i.e., cannot be classified elsewhere, such as Guyanese, Pacific Islanders, Polynesian, Tibetan, or West Indian) Multiple visible minorities (i.e., two or more mark-in responses associated with groups designated as visible minorities) Not a visible minority (i.e., either a single mark-in response of ‘White’ or a write-in response not associated with a group designated as a visible minority, such as Israeli, Polish, Scottish or Swedish) Indigenous status (i.e., First Nations, Métis or Inuk/Inuit)
USA [23]		
Standards for the Classification of Federal Data on Race and Ethnicity	Self-identified race and ethnicity: five minimum categories for race and two categories for ethnicity	Race: American Indian or Alaska Native Asian Black or African American Native Hawaiian or Other Pacific Islander White Ethnicity: Hispanic or Latino Not Hispanic or Latino (Possible ethnic groups could include Hispanic, non-Hispanic American Indian or Alaska Native, non-Hispanic Black, non-Hispanic White, etc.)
United Kingdom [28]		
National Census: England and Wales	Self-identified race and ethnicity. People are asked to choose their ethnic group (by ticking the option that best describes their ethnic group)	Asian or Asian British—Options are as follows: Indian Pakistani Bangladeshi Chinese Any other Asian background Black, Black British, Caribbean or African: Options are as follows: Caribbean African Any other Black, Black British, or Caribbean background Mixed or multiple ethnic groups—Options are as follows: White and Black Caribbean White and Black African White and Asian Any other mixed or multiple ethnic background White—Options are as follows: English, Welsh, Scottish, Northern Irish or British Irish Gypsy or Irish Traveller Roma Any other White background Other ethnic groups—Options are as follows: Arab Any other ethnic group, write in
Northern Ireland	Self-identified race and ethnicity. People are asked to choose their ethnic group (by ticking the option that best describes their ethnic group)	What is your ethnic group? Options are as follows: Black African Black Other Chinese Filipino Indian Irish Traveller Mixed ethnic group Roma White Any other ethnic group
Scotland	Self-identified race and ethnicity. People are asked to choose their ethnic group (by ticking the option that best describes their ethnic group)	Asian, Scottish Asian or British Asian—Options are as follows: Pakistani, Scottish Pakistani or British Pakistani Indian, Scottish Indian or British Indian Bangladeshi, Scottish Bangladeshi or British Bangladeshi Chinese, Scottish Chinese or British Chinese Other African, Scottish African or British African Respondents write in their ethnic group Caribbean or Black Respondents write in their ethnic group Mixed or multiple ethnic group Respondents write in their ethnic group White Scottish Other British Irish Polish Gypsy or Traveller Roma Showman or Show-woman Other Other ethnic group Arab, Scottish Arab or British Arab Other (for example, Sikh, Jewish)
Identity codes: Codes used by the British police (Still widely used)		Codes Asian or Asian British A1 Indian A2 Pakistani A3 Bangladeshi A9 Any other Asian background Black or Black British B1 Caribbean B2 African B9 Any other Black background Mixed M1 White and Black Caribbean M2 White and Black African M3 White and Asian M9 Any other mixed background Chinese or any other ethnic group O1 Chinese O9 Any other ethnic group White W1 British W2 Irish W9 Any other White background

* Non-Caucasian in race or non-white in colour other than Aboriginal peoples.

## Data Availability

Not applicable.
